# β-catenin and Its Relation to Alveolar Bone Mechanical Deformation – A Study Conducted in Rats With Tooth Extraction

**DOI:** 10.3389/fphys.2020.00549

**Published:** 2020-06-05

**Authors:** Beatriz Carmona Ferreira, Alexandre Rodrigues Freire, Rafael Araujo, Gleyson Kleber do Amaral-Silva, Roberta Okamoto, Felippe Bevilacqua Prado, Ana Cláudia Rossi

**Affiliations:** ^1^Laboratory for Mechanobiology Research, Biosciences Department, Piracicaba Dental School, University of Campinas, Piracicaba, Brazil; ^2^São Leopoldo Research Institute, São Leopoldo Mandic University, Campinas, Brazil; ^3^Oral Pathology Laboratory, Oral Diagnosis Department, Piracicaba Dental School, University of Campinas, Piracicaba, Brazil; ^4^Laboratory for Study of Mineralized Tissue, Basic Sciences Department, School of Dentistry of Araçatuba, São Paulo State University, Araçatuba, Brazil

**Keywords:** mechanobiology, rat, finite element analysis, beta-catenin (B-catenin), alveolar bone, mechanical deformation

## Abstract

The aim of this study was to analyze the relationship between alveolar bone deformation and β-catenin expression levels in response to the mechanical load changed by dental extraction in adult rats. Twenty-four male rats (*Rattus norvegicus albinus*), Wistar linage, at 2 months of age, were used. The right upper incisor tooth was extracted, and euthanasia occurred in periods 5 (*n* = 6), 7 (*n* = 6), and 14 (*n* = 6) days after Day 0. In the control group (*n* = 6), the dentition was maintained. The euthanasia occurred within 14 days after day 0. After euthanasia, the rats of all groups had their left jaw with tooth removed and separated in the middle. The pieces were undergone routine histological processing and then the immunohistochemical marking were performed to label expression of the primary β-catenin antibody, which was evaluated by qualitative and quantitative analysis. One head by each group (control and experimental) was submitted to computerized microtomography. After the three-dimensional reconstruction of the skull of the rat in each group, the computational simulation for finite elements analysis were performed to simulate a bite in the incisors. In finite element analysis, the strain patterns were evaluated after the application of bite force. The results were analyzed considering the areas in which changes in the amount of deformations were detected. The action of the bite force in the experimental condition, resulted in a uniform distribution of the amount of deformations, in addition to lower amount of deformation areas, differentiating from the control group. Comparing with the control group, the levels of β-catenin signaled in the lingual bone of the middle third of the alveolar bone were raised in the periods of 5 and 14 days. The increased β-catenin positive staining intensity was concentrated on osteocytes and gaps of osteocytes. The findings of the present study were in accordance with our hypothesis that the condition of dental extraction can cause the expression of β-catenin and alter the regimes of bone deformation.

## Introduction

Bone tissue is physiologically dynamic. It is in continuous remodeling cycle of bone formation and resorption, maintaining a constant calcium homeostasis and bone mass during vertebrates’ lives (Kobayashi et al., 2016). The periodontal ligament and the mineralized tissues support many functional loads and transmit them to alveolar bone. Therefore, the magnitude, the direction, the frequency and the duration of these loads determine alveolar bone remodeling (Beertsen et al., 1997; Ho et al., 2010).

Dental occlusal trauma plays an important role in alveolar bone remodeling and its effects includes many cytokines and signaling pathways. However, the exact mechanism of the traumatic stimulus for alveolar bone remodeling is still unclear (Wan et al., 2012). Changes in mechanical stimulation as a result of dental extraction can lead to changes in bone tissue. Traumatic occlusal forces are responsible for altering the expression of osteoblasts and osteoclasts genes, and thus may cause bone resorption (Walker et al., 2008).

Mechanobiology is responsible for studying the action of these mechanical changes on biological processes through cellular signaling, enabling the understanding of how these tissues are produced, maintained and adapted by the cells as a reply to mechanical stimuli. The research in this field can be divided into two fields: mechanoregulation algorithms to estimate changes in bone tissue and models that analyze cellular mechanobiology (van der Meulen and Huiskes, 2002; Giorgi et al., 2016).

Understanding of cell behavior and expression of proteins related to tissue changes is possible by means of immunohistological analysis (Lin et al., 2017). One critical and most studied Wnt pathway is the canonical Wnt signaling, that works by regulating the amount of the transcriptional co-activator β-catenin, which controls the major programs of gene expression in development (MacDonald et al., 2009).

It is established that Wnt plays an important role in the regulation of bone mass in humans and rodents, positively affecting bone formation by managing osteoblasts at different degrees, and it is recognized as an essential regulator of osteoblast activity through the translation of mechanical loading into biochemical signals (Baron and Rawadi, 2007; Tyrovola, 2019).

The Wnt signaling pathway is regulated by molecular and mechanical extracellular environment signals and its biological effects well settled (Robling and Turner, 2009). Wnt/β-catenin signaling activation induces osterix expression, a transcription factor that promotes osteoblast differentiation. Besides that, osteoprotegerin (OPG) is a target gene of this signaling pathway. Therefore, the expression of OPG is induced, when signaling is activated, in order to inhibit osteoclasts differentiation, preventing bone resorption (Kobayashi et al., 2016).

The literature (Bonewald and Johnson, 2008; Xu et al., 2016) cited the osteocytes as the major bone cell type responsible for mechanical strain sensing, acting in the bone remodeling process by sensing and transmitting external mechanical loading information to effector cells. The osteocytes use the intracellular Wnt/β-catenin pathway to transmit signals of mechanical loading to cells on the bone surface (Bonewald and Johnson, 2008).

The finite element analysis is an *in silico* evaluation that has been applied when studying living tissues, having a wide spectrum of clinical applications (Tsouknidas et al., 2015). The effectiveness of this analysis depends on obtaining precise models (Hohmann et al., 2011). However, it is now possible to obtain proper models, since methods such as computerized tomography or microtomography or serial sections of the samples can be used (Mehari Abraha et al., 2019). Its acceptance allowed the analysis to become vastly used in biomechanics, and the development of increasingly complex and sophisticated three-dimensional models made it easier the comprehension of the *in situ* mechanical responses of biological systems (Mehari Abraha et al., 2019).

Regarding alveolar bone, studies using the finite element method have shown that, whether in rodents, non-human primates or humans, there is a bone adaptation coming from different Strain regimes associated with chewing (Cox et al., 2012; Prado et al., 2016). Tooth absence can lead to occlusal hypofunction, that is a reduced bite force on the antagonist tooth and adjacent alveolar bone during mastication and, then, cause a negative impact in bone homeostasis (Shimomoto et al., 2007; Xu et al., 2016).

In this study, the right upper incisor tooth extraction was performed to cause altered mechanical loading to study the tissue response of the alveolar bone from the adjacent tooth (left upper incisor tooth), considering that the mastication force was altered. Thus, our hypothesis was that the tooth extraction condition can cause β-catenin expression and altered strain pattern of the alveolar bone of the adjacent tooth (left upper incisor). The aim of this study was to analyze the relationship between alveolar bone deformation and β-catenin expression levels in response to the mechanical load changed by tooth extraction in adult rats.

## Materials and Methods

This study was approved by the Ethics Committee on Animal Experimentation (CEUA) from the Biology Institute (IB) of the University of Campinas (UNICAMP) (protocol number: 4674-1/2017).

### Study Design

Twenty-four male rats (*Rattus norvegicus albinus*), Wistar linage, 2 months old (average body weight of 200–250 g), from CEMIB-UNICAMP were kept in collective cages (four animals/box), with temperature at 22 ± 2°C, controlled light cycle (12/12 h) and free access to water and feed. The rats were randomly assigned to different groups for the experiments:

– *Control group (n* = *6):* normal dentition was maintained. Euthanasia occurred at 14 days after day 0 of the exodontia.
– *Tooth extraction groups (n* = *18):* the rats had their right upper incisor extracted, and the euthanasia occurred at 5 (*n* = 6), 7 (*n* = 6), and 14 (*n* = 6) days after day 0 of the exodontia.

### Tooth Extraction

The procedure was performed under sedation via an intraperitoneal injection of ketamine (40–87 mg/kg), to promote anesthesia, and xylazine (5–13 mg/kg), to promote muscle relaxation. Once checked the sedation and anesthesia signals, the right upper incisor was extracted using specific and adapted instruments (Okamoto and de Russo, 1973). The gingival mucosa was sutured with polyglactin 910 (Vicryl 4.0 – Jhonson & Jhonson, New Brunswick, NJ, United States). After the surgery procedure, was promote analgesia using an injection of ketoprofen (NSAID – 5 mg/kg) via subcutaneous, one time, per 1 day.

The euthanasia of the animals was performed in the periods previously proposed for both the control and the experimental group due to excessive anesthetic dose. The head was disjointed of the body and dissected to obtain the skull and fixed in 10% formalin solution and 0.1 M phosphate buffer (pH 7.4) for 24 h at 4°C.

### Micro-CT Scanning Procedure

One specimen of the control group and one of the experimental group (14 day after extraction) were scanned in the Skyscan 1174 microtomography (Bruker, Belgium). The peak voltage was 50 kV and tube current was 800 μA. Image pixel size was 30 μm. After scanning, images were imported into NRecon Reconstruction software (SkyScan, Leuven, Belgium) for reconstruction in grayscale in the axial plane and presenting x-ray attenuation coefficients with values related to mineralized structures.

### 3D Model Preparation to Finite Element Analysis

**FIGURE 1:**
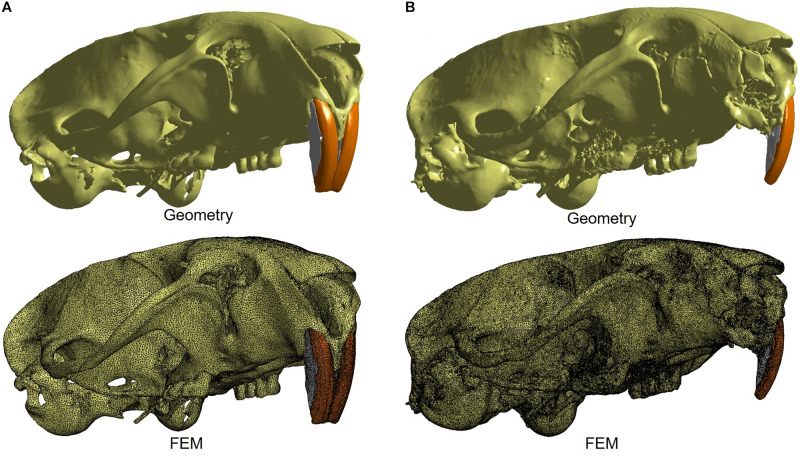
The 3D geometry and finite element model of control **(A)** and experimental **(B)** groups, which both are characterized by a mesh composed by tetrahedral elements. The image illustrates the model designed in the Materialise 3-Matic v10, which is associated with the Materialise MIMICS software.

The reconstructed micro-CT images were imported into Materialise Mimics Research v. 18 software (Materialise, Belgium) for segmentation of the structures required for the construction of the finite element model, which were: bone structure, periodontal ligament, enamel, dentin and pulp. All structures were converted to a 3D surface and, then, converted to finite element mesh (Figure 1). For the periodontal ligament and pulp, the 3D surfaces were constructed based on their spaces.

The finite element model was imported to software Ansys v. 17.2 (Ansys Inc., EUA) that was used to assign the mechanical properties of anatomical structures. The structures were assigned as linear elastic and isotropic (Table 1), whose values were determined experimentally in rats according to the study of Cox and Jeffery (2011); Cox et al. (2012).

**TABLE 1:** Mechanical properties of the anatomical structures considered in the study.

Structure of the tooth	Elasticity modulus (MPa)	Poisson’s ratio
Bone	19,920	0.3
Enamel	62,370	0.33
Dentin	23,600	0.31
Pulp	2	0.45
Periodontal ligament	50	0.4

*MPA, megapascal.*

### Finite Element Analysis Configuration

**FIGURE 2:**
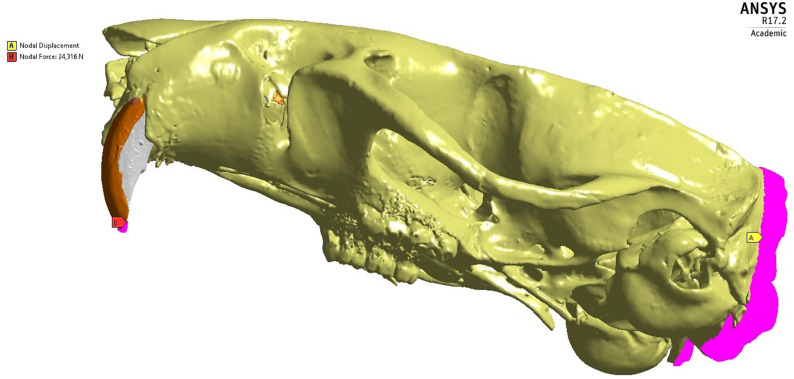
Model showing the nodes (pink) related to the boundary **(A)** and loading **(B)** conditions in Ansys software.

In order to simulate the biting force in the incisors for the control group (no tooth extraction) and experimental group (extraction of the right upper incisor), the analysis was configured for the action of the food break, that is the phase corresponding to the force of maximum bite, equal to 24.31N (Robins, 1977), for both groups. Restraints were applied on the posterior cut plane of the skull to keep the stability and absence of skull movements during the muscular action (Figure 2).

### Sample Preparation for Immunohistochemistry Analysis

After euthanasia, the rats of all groups had their left maxilla (with tooth) removed. The pieces were fixed in 10% formaldehyde for 24 h, washed for 24 h in running water and decalcified in EDTA 4.13% (Merck^®^, EMD Millipore Corporation, Germany) (Warshawsky and Moore, 1967; Arana-Chavez and Nanci, 2001) for 3 months at room temperature. Once the decalcification was verified, the pieces went through routine histotechnician processing. The obtained blocks were cut in Lupetec MRP03 microtome (Indústria Tecnológica de Equipamentos para Laboratório LTDA – ME, São Carlos-SP) in cuts of 5 μm thickness and mounted on silanized blades.

For the immunohistochemical reactions, the activity of the endogenous peroxidase was inhibited with hydrogen peroxide. Then, the slides underwent the antigenic recovery step with phosphate citrate buffer (pH 6.0, heated 55°C, 40 min). The primary antibody used was β-catenin/Wnt pathway, polyclonal (Anti-beta Catenin antibody ab6302 – Abcam – United States). The secondary antibody used was Universal Polimere (N-Histofine^®^ Simple Stain Rat max po multi - NICHIREI BIOSCIENCES), and diaminobenzidine (Dako) as chromogen.

The slides were scanned using the Aperio Digital Pathology System (Aperio Technologies Inc., Vista, CA, United States) and the images were captured in the Aperio ImageScope Pathology Slide Viewing Software (Leica Biosystems Inc., Buffalo Grove, IL, United States) centralizing the middle third of the alveolus at a magnification of 40×.

### Data Analysis

**FIGURE 3:**
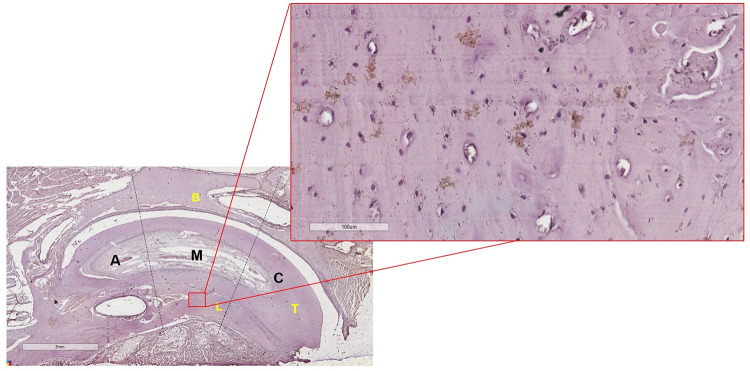
Scheme showing the selection of the middle third of the alveolus in a slide (magnification of 1.25x) of the left upper incisor. In lingual alveolar bone of the middle third of the alveolus, the selection of the area of interesting was at a magnification of 40× to perform the cell count. A, Apical third of the left upper incisor; M, Middle third of the left upper incisor; C, Cervical third of the left upper incisor; B, Buccal alveolar bone; L, Lingual alveolar bone; T, Tooth.

The middle third of the alveolus of the left upper incisor was evaluated (Figure 3). Both analyses were directed to the same region to make it possible to relate the results obtained in Strain analysis to those obtained in the analysis of beta-catenin expression, proposed in this study.

#### Finite Element Analysis

Bone deformation patterns were evaluated in the alveolar bone adjacent to the upper incisor, after the application of bite force. The results were obtained by calculating the amount of deformation (equivalent strain). The results were analyzed considering the areas in which changes in the amount of deformations were detected (ε). The analysis of the control group served as a parameter to characterize the normal distribution of the amount of deformations during the phase of maximum bite force required in the feed. Furthermore, the color scale with the strain value intervals was configured to visualize a general feature and for alveolar bone region.

#### Qualitative Analysis of Immunohistochemistry

The analysis was performed according to the visualization of the intensity of chromogen marking in the osteocytes (Figure 3), in the osteocyte gap and in the periphery of the osteocytes. The 40× magnification was used.

#### Quantitative Analysis of Immunohistochemistry

The quantitative analysis of the positive beta-catenin immunostaining cells was performed in ImageJ Software (National Institutes of Health - Public Domain), using the images that were captured in the Aperio ImageScope Pathology Slide Viewing Software, centralizing the middle third of the alveolus at a magnification of 40× (Figure 3). Three stained slides with one tissue section each (using semi seriated sections) from left upper incisor with alveolus per group were used for evaluation. Thus, three sections of the interest area were used for cell counting, totalizing 12 slides. The method used was the manual cell counting (positives osteocytes presented beta-catenin expression) (plug-in: cell counter). We only counted the osteocytes with positive staining. The counted cells were expressed as a percentage of the total number of cells (Lara-Castillo et al., 2015).

### Statistical Analysis

The results obtained in the quantitative analysis of immunohistochemistry were submitted to statistical analysis. For evaluation of the parametric data distribution the Shapiro-Wilk test was used. The data were submitted to the ANOVA test to verify the difference between the means of the percentages obtained in the cell count and the Tukey *post hoc* test to detect differences in the comparison between the groups (Software R – R CRAN SOURCE). The level of significance of *p* < 0.05 was considered.

## Results

### Finite Element Analysis

**FIGURE 4:**
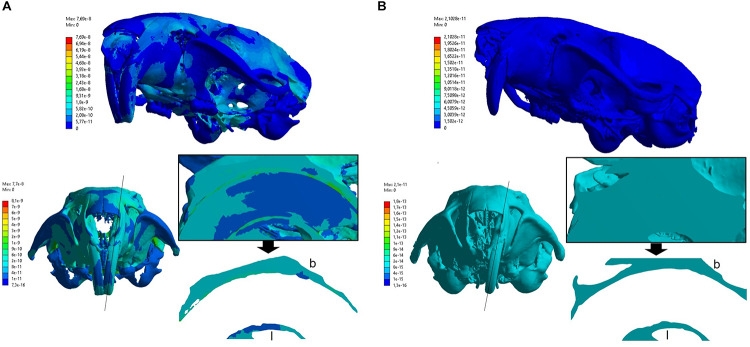
Amount of deformations (strain) distribution in the control group **(A)** and experimental group **(B)**. The top part of the figure shows the general color scale for the strain distribution. The bottom part shows the modified color scale configured for alveolar bone region. The black line indicates a sagittal cut plane, which the middle third of the alveolar region was highlighted in the black border square (containing the bone, periodontal ligament, and tooth). Below the square, the buccal (b) and lingual (l) alveolar bone lamina were isolated for better view. Note the experimental group presented a uniform color strain distribution, being difficult to identify the structures in the sagittal view. **(A)** Control group. **(B)** Experimental group.

In the control group, it can be observed a non-uniform distribution with medium strain magnitude areas ranging from 0.0001 to 0.001 με in the alveolar bone. These areas are in both buccal and lingual alveolar bone at the middle third of the alveolus, wherein the buccal bone presented higher strain (Figure 4). In addition, Figure 4A shows higher strain magnitude in the periodontal ligament (ranging from 0.002 to 0.03 με).

In the experimental group, the action of the bite force resulted in distribution of lower strain magnitude than the control group, ranging up to 6 × 10^–8^ με (Figure 4) in the alveolar bone. The strain magnitude in this group was low in both buccal and lingual bone at the middle third of the alveolus.

### Qualitative Analysis of Immunohistochemistry

**FIGURE 5:**
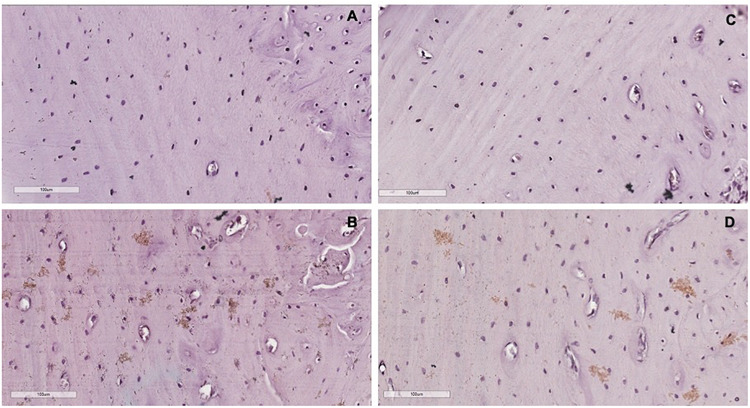
Representative cuts of each group in the 40× objective. **(A)** Control group. **(B)** 5 days group. **(C)** 7 days group. **(D)** 14 days group. The brown areas represent the staining of β-catenin by the chromogen. Contra-staining: Hematoxylin.

Comparing with the control group, we observed that the levels of β-catenin signaled by diaminobenzidine staining areas (brown areas) in the lingual bone of the middle third of the alveolus were raised in the periods of 5 and 14 days. We have found that this increased beta-catenin positive staining intensity was concentrated on osteocytes and gaps of osteocytes. Representative sections of each group on the 40× objective can be seen in Figures 5A–D. Supplementary Data Sheet 1 shows the representatives cuts of the groups in the 20x objective.

### Quantitative Analysis of Immunohistochemistry

**FIGURE 6:**
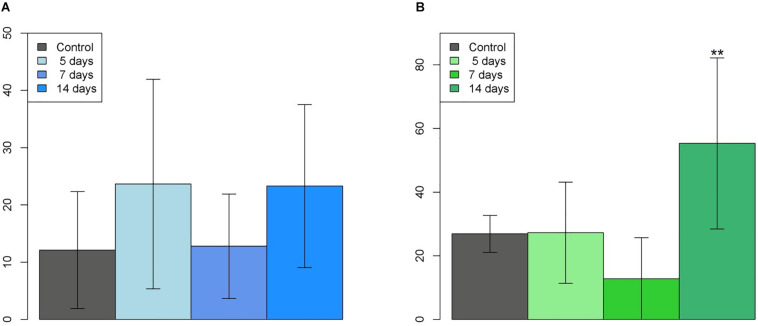
Graphs of the cell count in the **(A)** Buccal (ANOVA: *p* = 0.288) and **(B)** Lingual (ANOVA: *p* = 0.00303**) alveolar bone of the left upper incisor.

For the buccal alveolar bone lamina of the left upper incisor, Table 2 shows the mean osteocyte cell counts with positive β-catenin staining in each group. ANOVA showed no significant differences between groups (*p* = 0.288), as it can be observed in Figure 6.

**TABLE 2:** Mean values of positive markings in each group studied (buccal alveolar bone lamina).

Group	Mean (%)	Standard deviation	Shapiro-Wilk (*p*-value)
Control	12.11	10.21	0.7070255
5 days	23.65	18.29	0.5391761
7 days	12.78	9.11	0.755004
14 days	23.30	14.21	0.3760958

For the lingual alveolar bone lamina of the left upper incisor, Table 3 shows the mean osteocyte cell counts with positive β-catenin staining in each group. ANOVA showed that there were significant differences when we observed the group 14 days compared to all the other groups (*p* = 0.00303^∗∗^), as it can be observed in Figure 6.

**TABLE 3:** Mean values of positive markings in each group studied (lingual alveolar bone lamina).

Group	Mean (%)	Standard deviation	Shapiro-Wilk (*p*-value)
Control	26.91	5.80	0.8599952
5 days	27.26	15.88	0.2944084
7 days	12.78	12.94	0.3723958
14 days	55.33	26.86	0.8554278

Table 4 shows the values corresponding to the comparisons between the groups on the lingual alveolar bone lamina of the left upper incisor, and the Tukey *post hoc* test detected a significant difference for comparison of the 14 days (d) group with the other groups.

**TABLE 4:** Values of the comparisons between the groups on the lingual alveolar bone lamina of the left upper incisor by the Tukey *post hoc* test.

Relation	Diff	Lwr	Upr	*p*-value
5d-control	0.35000	−27.3536322	28.05363	0.9999835
7d-control	−14.13333	−41.8369655	13.57030	0.4974262
14d-control	28.41667	0.7130345	56.12030	0.0431173
7d-5d	−14.48333	−42.1869655	13.22030	0.4769335
14d-5d	28.06667	0.3630345	55.77030	0.0463788
14d-7d	42.55000	14.8463678	70.25363	0.0018300

*Diff, difference in the observed means; Lwr, lowe end point of the interval; Upr, upper end point.*

## Discussion

Animal models provide relevant information about a variety of disorders in dentistry. The rat model has been widely used for studies in dentistry, including those which involve periodontal diseases, alveolar bone healing, tooth movement, osteogenesis, osseointegration, use of biomaterials, and altered occlusal loadings, for example (Okamoto and de Russo, 1973; Shimomoto et al., 2007; Walker et al., 2008; Wan et al., 2012; Xu et al., 2016; Zeng et al., 2016; Koutouzis et al., 2017; Lee et al., 2017; Hassumi et al., 2018; Palin et al., 2018; Uslu et al., 2018; Wang et al., 2018). Besides that, tooth extraction, inclosing incisor extraction, has been considered as a classical model of preclinical studies and has been applied in many studies with different aims in the last years (Avivi-Arber et al., 2010; Shoji et al., 2010; Boonyagul et al., 2014; Curra et al., 2016; Hassumi et al., 2018; de Oliveira Puttini et al., 2019).

Medical and dental science has benefited from the rat as a model through the investigation of pathological conditions affecting craniofacial morphology. As the rat has in the past, and continues to play, such a crucial role in the advancement of understanding of craniofacial development, morphology and evolution, it is surprising that very little published data exists regarding the anatomy, and in particular the craniofacial biomechanics behavior.

Mechanobiology is governed by cells response within the tissue to mechanical forces, and is essential to bone adaptation, since the skeletal tissues morphology and structural fitness are modulated by mechanical forces. Mechanically load-induced strains in the cells and stresses of fluid flow can bring on changes in gene expression in bone tissue. It is known that bone is a complex tissue made up of cells and collagenous matrix, which includes minerals, and its homeostasis is strongly conditioned on mechanosensory regulatory signals of environmental forces in biochemical responses. Bone architecture and mass modifying occurs to make possible a more efficient performance of the bone tissue. It is clear that bone cells act as mechanosensors, but the central issue in this study field is how external muscle loads are to skeletal tissues, how bone cells perceive these loads, and how these signals are translated into biochemical reactions to produce cell expression or differentiation, resulting in macroscopic changes in the bone structure (van der Meulen and Huiskes, 2002; Feller et al., 2015; Ruggiu and Cancedda, 2015).

According to the results of the present study, it is believed that the incisor tooth adjacent to the extracted tooth can present a modified masticatory loading regime. It is worth mentioning that the scarcity of information on how the rat behaves *in vivo* in chewing after extraction should be considered.

Little is known about the transduction of osteogenic signals to stimulate bone formation or bone resorption, because this pathway is hard to illustrate. Nevertheless, some recent molecular advances allow looking for genes and molecules to understand their role in the adaptation pathways (van der Meulen and Huiskes, 2002). Thus, the present study had the aim to evaluate Wnt/β-catenin signaling in bone, once this protein is responsible for the transmission of mechanical stimuli to biological signals for bone formation.

It is accepted that mechanical loads placed on bone generate a variety of stimuli that could be detected by the osteocyte, but the mechanism by which osteocytes sense mechanical strain has yet to be fully understood and described (Oftadeh et al., 2015). The Wnt/β-catenin pathway has been identified using *in vitro* and *in vivo* approaches to be an important pathway in osteocytes to sense and transduce the signals of mechanical stimuli to bone cells (Duan and Bonewald, 2016). Activation of Wnt/β-catenin signaling was related as a normal response to bone stimulation (Robinson et al., 2006; Javaheri et al., 2014; Lara-Castillo et al., 2015).

In the present study, when we induced the change in the normal stimulus, i.e., extracted the right upper incisor, we observed the activation of Wnt/β-catenin signaling in alveolar bone. We can suggest that the bone cells mechanosensing by an altered loading pattern microenvironment regulate the bone tissue behavior coordinating adaptive alterations in bone mass and architecture. In response to unusual signals several mechanotransduction pathways are activated (Shivashankar et al., 2015; Giorgi et al., 2016).

In the present study, the mechanical load pattern was altered by tooth extraction. It was possible to note the Wnt/β-catenin signaling in response to this altered load pattern, as it can be seen by a greater positive β-catenin staining osteocyte of the lingual bone in the 14-day group. β-catenin signaling in the alveolar bone of the adjacent tooth allows the understanding that the bone tissue had to adapt its structure to these new loads that were applied. Lara-Castillo et al. (2015) observed that β-catenin signaling activation in osteocytes occurred in regions that suffered both compression and tension after a mechanical stimulus. They suggested that the critical factor is the magnitude of the change, not necessarily the type of deformation applied.

To date, little is exposed about how β-catenin signaling is physiologically modulated by masticatory loads (Galli et al., 2010; Oftadeh et al., 2015). It is necessary developing additional studies to verify if the β-catenin activation pattern corresponds spatially and temporally with bone remodeling against mechanical stimuli. In the literature, it has been consistent that β-catenin plays an important role in bone formation and remodeling (Herencia et al., 2016).

Frost introduced the “mechanostat theory” in bone biology to understand the “minimum effective strain (MES),” which relates bone architecture changes to mechanical loads (Tyrovola, 2019). In our study, the FEA was used in order to figure out about the bone tissue threshold for strain associated with the β-catenin activation in alveolar bone, and how is the behavior of the alveolar bone after induction of changes in mechanical stimulus on adjacent tooth (upper left incisor) due to tooth extraction. We verified that the alveolar bone was deformed with changes in mechanical strain in comparison of two group.

The computational simulation by FEA in this present study has the limitation being a static analysis, presenting only the strain distribution from a bite load. In addition, we predicted that the experimental group apply the same bite force during the power stroke phase. In fact, dynamic features must be considered in a future study, as the frequency of bite loads as well as changes in the force orientation in result of a possible changes in the mandible position during incisor biting. Thus, beyond to establish the relation between the β-catenin expression to the changes in the mechanical strain distribution, this relationship could include dynamic features during feeding.

In conclusion, the findings of the present study were in accordance with our hypothesis that the condition of tooth extraction can cause the expression of β-catenin and alter the regimes of bone deformation.

## Data Availability Statement

All datasets generated for this study are included in the article/Supplementary Material.

## Ethics Statement

The animal study was reviewed and approved by Ethics Committee on Animal Experimentation (CEUA) 4674-1/2017.

## Author Contributions

BF, AR, and AF contributed to conception of the manuscript. AR and BF contributed to the experiment. BF, AF, RA, GA-S, RO, and AR contributed to the data acquisition. BF, AR, FP, and AR contributed to the analyses and interpretation of the results. All authors contributed to design of the experiments and reviewed the manuscript.

## Conflict of Interest

The authors declare that the research was conducted in the absence of any commercial or financial relationships that could be construed as a potential conflict of interest.
