# Supplementary material for: β-catenin and Its Relation to Alveolar Bone Mechanical Deformation – A Study Conducted in Rats With Tooth Extraction
Source: Front Physiol. 2020 Jun 5;11:549. doi: 10.3389/fphys.2020.00549 (PMC7291952; doi:10.3389/fphys.2020.00549)
Supplement: Supplementary file 1 [file Data_Sheet_1.DOCX]

Supplementary Material

**
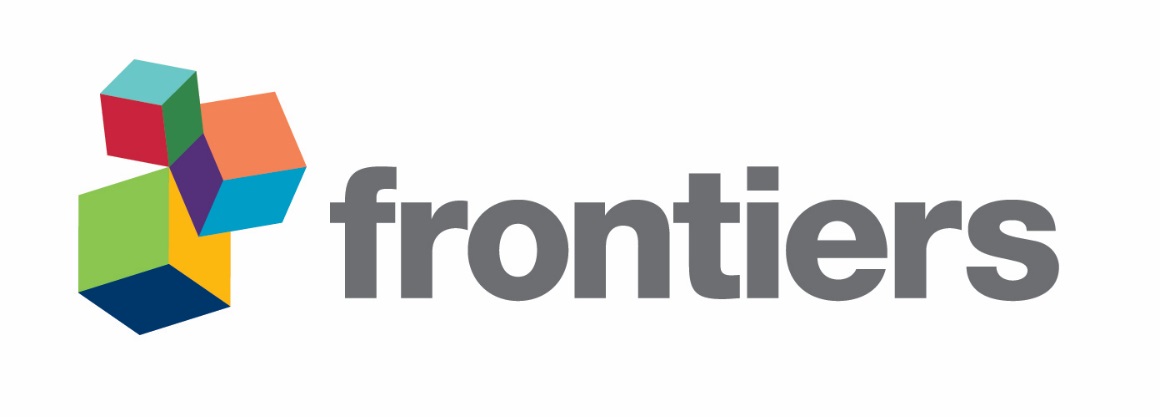
**

**Supplementary Figure 1.** The figure legends are required to have the same font as the main text, 12 point normal Times New Roman, single spaced. Please use a single paragraph for each legend and prepare the figures keeping in mind the PDF layout.


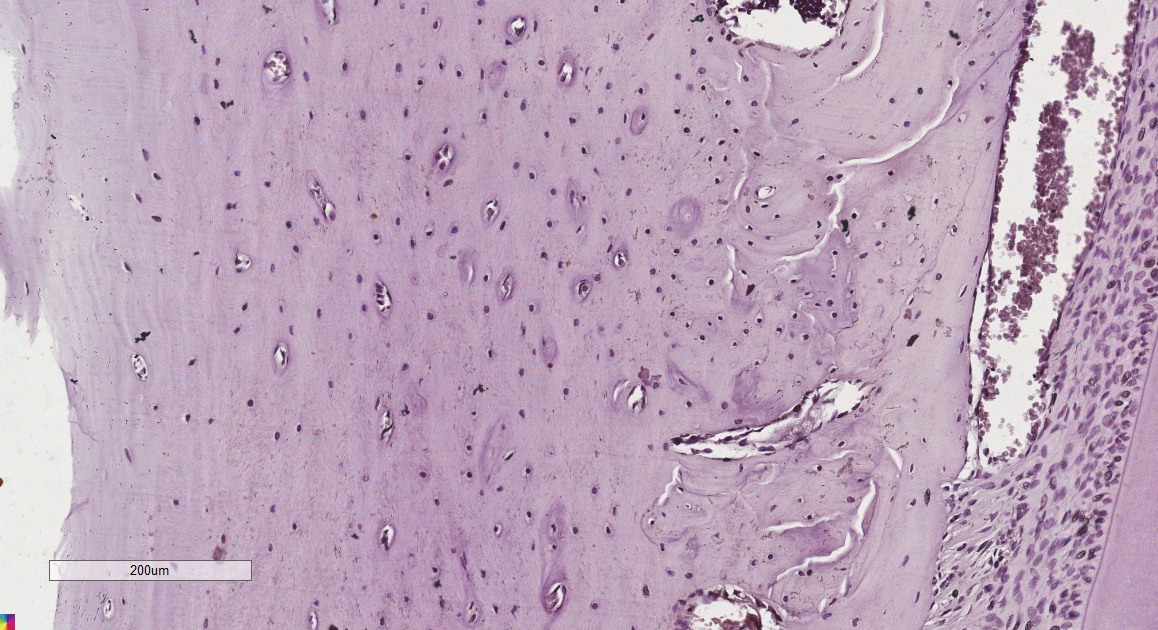


**Supplementary Figure 1.** Representative cut of the control group in the 20x objective. The brown areas represent the staining of β-catenin by the chromogen. Contra-staining: Hematoxylin.


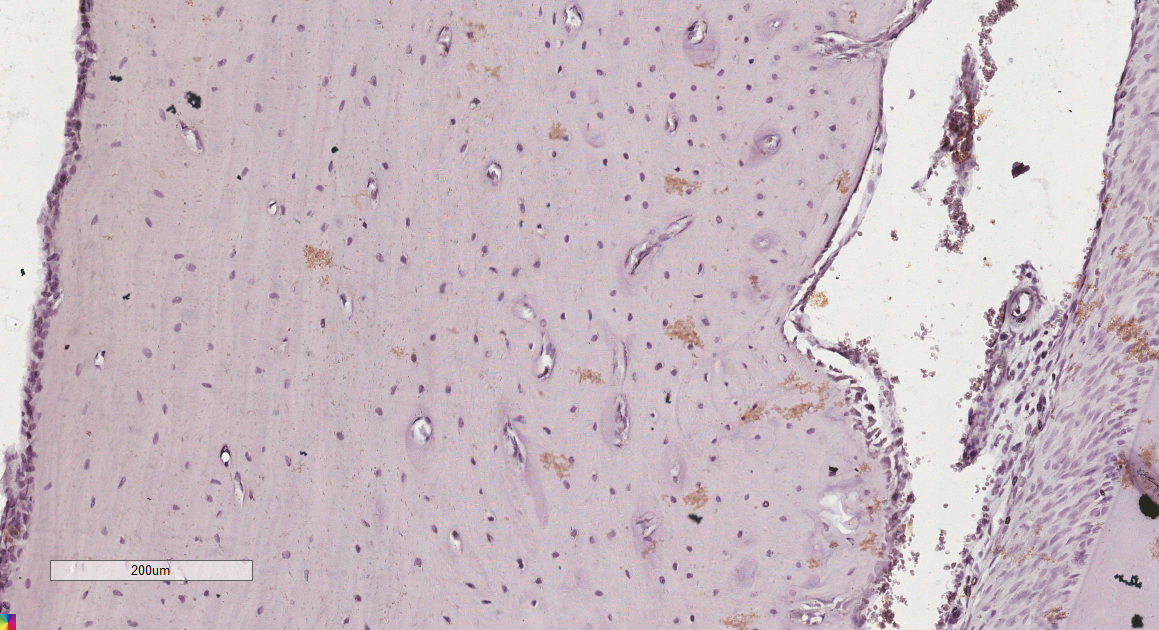


**Supplementary Figure 2.** Representative cut of the 5 days group in the 20x objective. The brown areas represent the staining of β-catenin by the chromogen. Contra-staining: Hematoxylin.


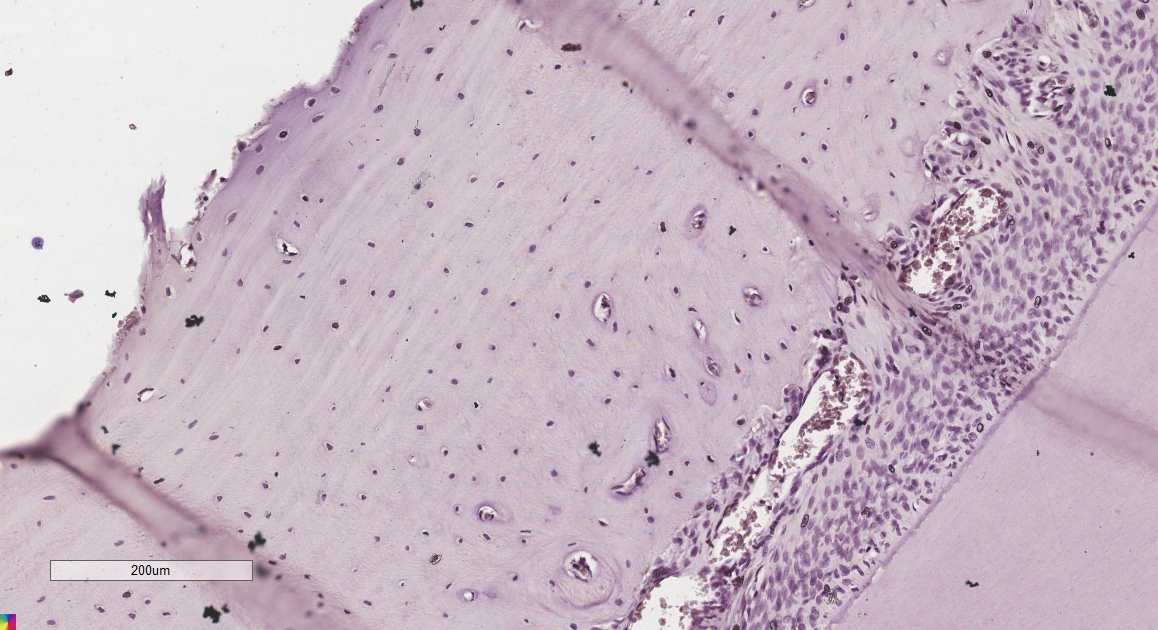


**Supplementary Figure 3.** Representative cut of the 7 days group in the 20x objective. The brown areas represent the staining of β-catenin by the chromogen. Contra-staining: Hematoxylin.


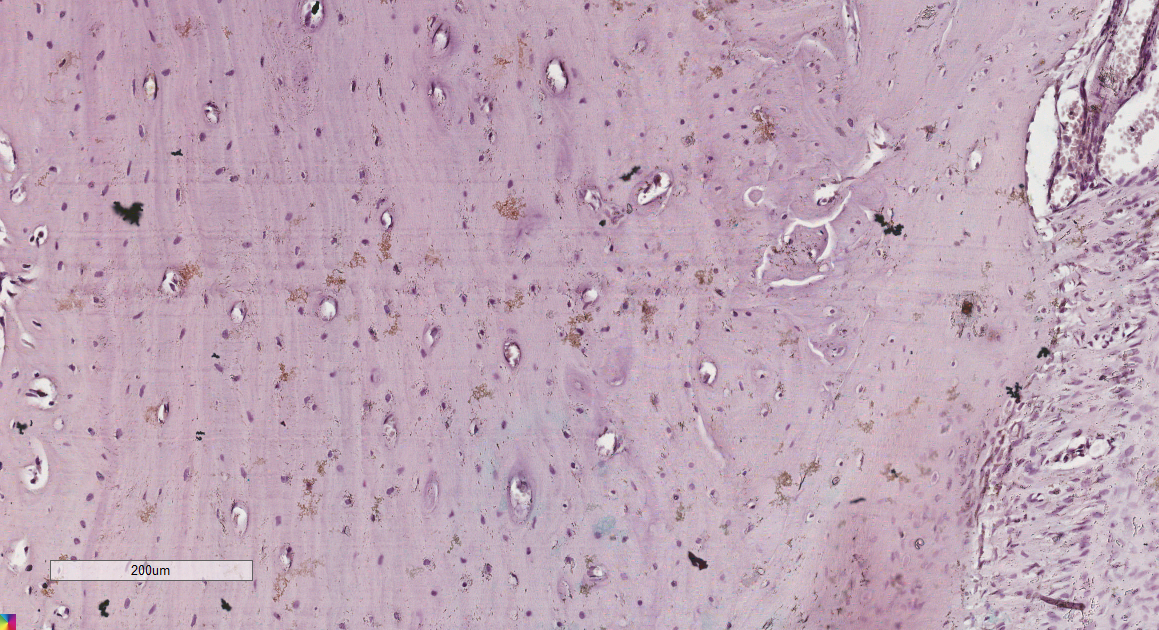


**Supplementary Figure 4.** Representative cut of the 14 days group in the 20x objective. The brown areas represent the staining of β-catenin by the chromogen. Contra-staining: Hematoxylin.
